# Nanobodies effectively modulate the enzymatic activity of CD38 and allow specific imaging of CD38^+^ tumors in mouse models *in vivo*

**DOI:** 10.1038/s41598-017-14112-6

**Published:** 2017-10-30

**Authors:** William Fumey, Julia Koenigsdorf, Valentin Kunick, Stephan Menzel, Kerstin Schütze, Mandy Unger, Levin Schriewer, Friedrich Haag, Gerhard Adam, Anna Oberle, Mascha Binder, Ralf Fliegert, Andreas Guse, Yong Juan Zhao, Hon Cheung Lee, Fabio Malavasi, Fernando Goldbaum, Rob van Hegelsom, Catelijne Stortelers, Peter Bannas, Friedrich Koch-Nolte

**Affiliations:** 1 Institute of Immunology, University Medical Center Hamburg-Eppendorf, D-20246 Hamburg, Germany; 2 Department of Radiology, University Medical Center Hamburg-Eppendorf, D-20246 Hamburg, Germany; 3 Department of Oncology and Hematology, University Medical Center Hamburg-Eppendorf, D-20246 Hamburg, Germany; 4 Department of Biochemistry and Molecular Cell Biology, University Medical Center Hamburg-Eppendorf, D-20246 Hamburg, Germany; 5 School of Chemical Biology and Biotechnology, Peking University Shenzhen Graduate School, Shenzhen, 518055 China; 6 Lab of Immunogenetics, Department of Medical Sciences, University of Torino Medical School, I-10126 Torino, Italy; 7 Fundacion Instituto Leloir, C1405 Buenos Aires, Argentina; 8 Ablynx nv, B-9052 Zwijnaarde, Belgium

## Abstract

The cell surface ecto-enzyme CD38 is a promising target antigen for the treatment of hematological malignancies, as illustrated by the recent approval of daratumumab for the treatment of multiple myeloma. Our aim was to evaluate the potential of CD38-specific nanobodies as novel diagnostics for hematological malignancies. We successfully identified 22 CD38-specific nanobody families using phage display technology from immunized llamas. Crossblockade analyses and in-tandem epitope binning revealed that the nanobodies recognize three different non-overlapping epitopes, with four nanobody families binding complementary to daratumumab. Three nanobody families inhibit the enzymatic activity of CD38 *in vitro*, while two others were found to act as enhancers. *In vivo*, fluorochrome-conjugated CD38 nanobodies efficiently reach CD38 expressing tumors in a rodent model within 2 hours after intravenous injection, thereby allowing for convenient same day *in vivo* tumor imaging. These nanobodies represent highly specific tools for modulating the enzymatic activity of CD38 and for diagnostic monitoring CD38-expressing tumors.

## Introduction

CD38 is a 43 kDa type II transmembrane ecto-enzyme that is highly expressed in hematological malignancies including multiple myeloma^1,2^. CD38 consists of a short intracellular N-terminal domain, a transmembrane helix and a long C-terminal extracellular catalytic domain^3^. CD38 is a multifunctional enzyme that catalyzes the synthesis of cyclic ADP-ribose and ADP-ribose from extracellular NAD^+^^4,5^. The presence of CD38 is routinely used as a marker for chronic lymphocytic leukemia (CLL) where high levels of CD38 correlates with a poor prognosis^6^. Binding of CD38 to its ligand CD31 enhances the proliferation and mirgration of CLL cells^6,7^. It has been proposed that the enzymatic activity of CD38 contributes to a microenvironment favorable for tumor survival in the bone marrow niche^8^. CD38 represents a promising target for monoclonal antibody (mAb)-based immunotherapy of multiple myeloma^9–12^. Daratumumab, a human IgG1 antibody generated by immunization of a human IgH-transgenic mouse, has shown promising results^13,14^. However, the use of mAbs has disadvantages that include the limited tissue penetration due to their large size of approximately 150 kD^15,16^

Nanobodies are the smallest antigen-binding domains derived from naturally occurring heavy chain antibodies from camelids. These single domain antibodies have several advantages over conventional antibodies, including the capacity to bind and block functional epitopes such as the active site cleft of enzymes, better tissue penetration *in vivo*, and the facile construction of bi- or multi-specific biologicals by genetic fusion^15,17,18^.

Binding of Daratumumab to CD38 prevents subsequent binding of many commercially available antibodies, which makes them unsuitable for plasma and myeloma cell identification in patients treated with daratumumab^19^. Thus, nanobodies that bind independently of daratumumab might be useful as companion diagnostic for identifying CD38 expressing cells in patients treated with daratumumab. Moreover, the enzymatic activity of CD38 may contribute to immune suppression observed in myeloma patients and to a microenvironment that is favorable for tumor survival in the bone marrow niche^8^. Thus, nanobodies that modulate the enzymatic activity of CD38 could have a therapeutic application for counteracting the immunosuppressive and tumor promoting activities of CD38.

Our goal was to evaluate the potential of CD38-specific nanobodies as novel diagnostics for hematological malignancies. Using phage display technology we successfully generated human CD38-specific nanobodies from immunized llamas. We characterized these nanobodies in terms of CD38-binding on cells, modulation of enzymatic activity, affinity, epitope specificity, complementary binding to daratumumab and targeting of CD38-expressing tumors in a mouse model. The results suggest that some of these nanobodies hold promise for detecting and monitoring CD38-expressing tumors.

## Results

### Panning of VHH-phage display libraries from immunized llamas on CD38-transfected cells yields 22 distinct families of CD38-specific nanobodies

Two llamas were immunized with recombinant nonglycosylated CD38 ecto-domain (aa 46–300) and two llamas were immunized with a cDNA expression vector encoding full length CD38 (Figure S1). Phage display libraries were generated by PCR-amplification of the VHH-repertoire from blood lymphocytes obtained 4–10 days after the last boost immunization^18,20^. CD38-specific nanobodies were selected by binding of phages to CD38-transfected lymphoma cells. Selected clones were sequenced and clones that were found more than once, or a plurality of clones with one or a few amino acid substitutions in the CDR regions, were defined as a family. The results revealed selection of clones derived from 22 distinct nanobody families, with CDR3 lengths ranging from 3 to 21 amino acid residues. FACS analyses performed with crude periplasmic lysates from *E. coli* to detect nanobodies that bound to CD38-transfected but not to untransfected cells, confirmed the specificity of the selected nanobody families for CD38 (Figure S2a).

Table 1 provides an overview of the CD38-specific nanobodies. For each family, the number of isolates (ranging from 1–27) and the number of variants within a family (ranging from 1–6) and the variant amino acid positions within the CDR3 region are indicated. Some nanobodies showed only little if any intrafamily variation, while others contained members with highly divergent amino acid sequences. Families 5, 14, and 20 contain the three nanobodies (MU375, MU1053, MU551) described in our previous study reporting the 3D-structures of these nanobodies in complex with CD38^21^.

**Table 1:** Characteristics of CD38-specific nanobody families.

**family**	**llama**	**isolate**	**variant**	**max diff**	**CDR3**	**name**	**clone**	**Kdis s** ^**−1**^	**epitope**
**1**	538	3	2	1	AHTFSGSF	l-8.1b	WF9	2.2E-03	3
**2**	539	6	6	10	*D*HTF*A*G*V*Y	l-8.2a	JK36	2.0E-04	3
**3**	539	1	1	0	VIRTYSTY	l-8.3a	WF42	3.8E-04	3
**4**	538	4	4	8	WHYAAGRDY	l-9.1c	JK2	1.1E-03	2
**5**	25	4	2	11	AS*T*AVGADT	l-9.2a	MU370	5.1E-04	1
**6**	538	2	2	8	GNPGT*R*Y*I*Y	l-9.3b	JK29	1.8E-04	1
**7**	538	2	2	3	DRFSVVAVEYDY	l-12b	WF14	6.3E-03	3
**8**	539	3	3	2	GLKRIGDQREADY	l-13a	JK28	6.3E-04	1
**9**	10	13	5	14	DRF*VV*AA*G*THDLDY	s-14a	MU738	2.9E-03	1
**10**	538	1	1	0	DRFTLVPTTSDLDY	l-14.1a	JK44	2.4E-04	1
**11**	539	4	4	7	R*F*WI*G*VRAPAEYNY	l-14.2b	JK22	3.2E-03	1
**12**	25	3	3	39	DR*LVLVA*LSIA*D*P*GF*	l-15.1b	MU1068	2.1E-04	1
**13**	25	27	2	21	GFPVL*V*AL*S*IADPDY	l-15.2a	MU274	1.1E-04	1
**14**	10	9	1	0	GRGIVAGRIPAEYAD	l-15.3a	MU1053	9.0E-04	1
**15**	539	1	1	0	GRTASASTMIREYDS	l-15.4a	JK19	1.1E-04	3
**16**	10	9	2	2	*T*TSVVVLLAPNWYEY	l+/−15a	MU415	1.1E-03	1
**17**	538	1	1	0	RLRGWITTRKPNEYDY	s-16a	WF211	4.5E-03	1
**18**	25	4	3	13	ARSA*G*LGSSRR*IE*GYD*K*	l-17a	WF121	2.3E-04	3
**19**	25	9	2	20	QYQ*D*RYY*DE*F*TW*KEK*D*M*DY*	l-19.1a	MU523	7.8E-05	2
**20**	25	3	2	6	RYQPRYYDSGDMDGYEY*EF*	l-19.2a	MU1067	1.2E-04	2
**21**	25	2	2	14	ADRFRGWATWRDDPDQYDY	s-19b	WF139	2.2E-04	3
**22**	10	6	6	13	DVTLNPFTGW*D*TRSGPMYRYEYDY	s+/−24a	WF100	5.1E-04	3
**Daratumumab**			VL: RSN		VH: DKILWFGEPVFDY	c-13	Dara scFv	4.4E-03	1

Families were designated in order of increasing CDR3 lengths. Isolate indicates the number of clones selected per family, variant indicates the number of clones carrying distinct but evidently related amino acid sequences, max diff indicates the maximum difference between two members of a family in number of amino acid substitutions. Variant amino acid positions in the CDR3 within a family are indicated in italic. Names indicate the presence of a short (s) or long hinge (l), the absence (−) or presence (+) of a disulfide bond connecting CDR2 and CDR3, and the length of the CDR3 in numbers of amino acid residues. Kdis shows off-rates determined by SPR analyses on the immobilized glycosylated extracellular domain of CD38. Epitopes are numbered arbitrarily, with nanobodies that block the binding of one another considered to recognize the same or overlapping epitopes.

### Characterisation of monovalent CD38-specific nanobodies carrying a C-terminal His6-c-Myc tag

For each nanobody family, we subcloned the member that had shown the highest staining intensity of CD38-transfected cells in the periplasmic screening assay (Figure S2b). To circumvent the problem of endotoxin contamination of nanobodies inherent to the *E. coli* expression system, we recloned the nanobody encoding region into a eukaryotic expression vector (pCSE2.5) optimized for secretory protein production in suspension cultures of HEK-6E cells in serum free medium^22–24^. SDS-PAGE analyses of HEK cell culture supernatants harvested 6d after transfection revealed consistent production levels of ~50 µg nanobody per ml of HEK-6E supernatant (Figure S3).

Specific binding of purified CD38 nanobodies were determined by off-rate analysis on real time bio-layer interferometry **(**BLI) analysis using the immobilized ectodomain of human CD38 (Table 1), revealing dissociation rates ranging from 7.8 × 10^−5^ to 6.5 × 10^−3^ s^−1^. Several nanobodies had very slow off-rates below the detection limit of the instrument (WF121, WF139, MU1105 and WF124). As reference, the single chain variable fragment (scFv) of Daratumumab (see below) was included (kd of 4.4 × 10^−3^ s^−1^). In addition, qualitative comparisons of the dissociation rates were performed using fluorochrome-conjugated CD38 nanobodies bound to CD38-transfected cells by flow cytometry over a timeframe of 16 hours (Figure S4). The results confirm the strong binding and slow dissociation from native CD38 on the cell-surface by monovalent CD38-specific nanobodies.

### Three nanobody families inhibit and two nanobody families stimulate the enzymatic activity of CD38

**Figure 1:**
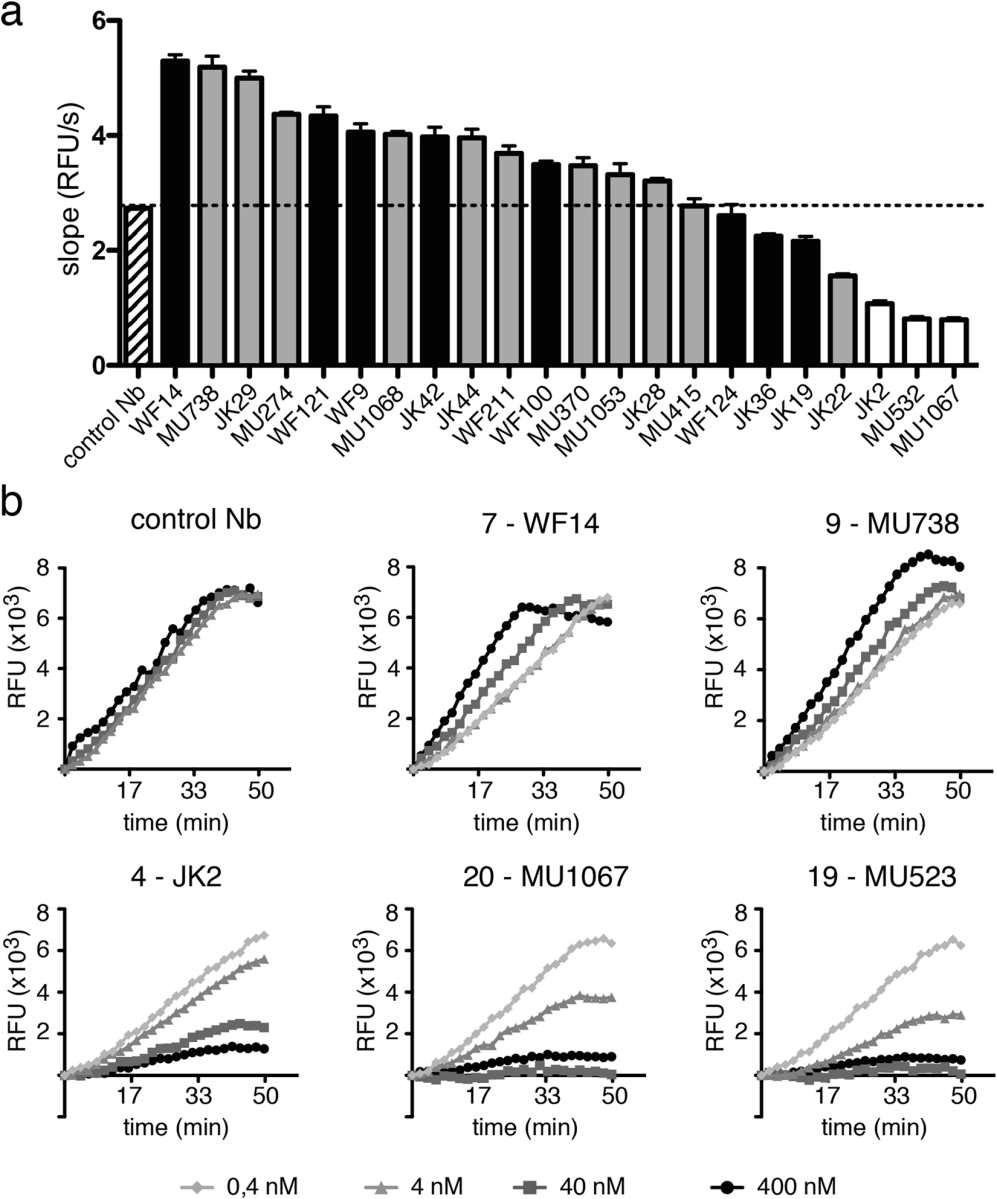
Identification of nanobodies that inhibit or enhance the enzymatic activity of CD38. (**a**) Recombinant human CD38 (5 nM) was incubated in the presence of CD38-specific nanobodies (400 nM) with NGD^+^ at RT. Production of cyclic GDP-ribose was monitored by fluorimetry (RFU = relative fluorescence units). Bars indicate the slope of the curves during the linear phase, e.g. from t = 10 min to t = 20 min) (n = 3). Bars are color coded according to the epitopes recognized by the respective nanobodies (grey = epitope 1, white = epitope 2, black = epitope 3, see Table 2). (**b**) Curves are shown for the enzymatic activity of CD38 (5 nM) in the presence of the two strongest agonistic nanobodies (WF14, MU738) and the two strongest antagonistic nanobodies (JK2, MU1067, and MU523), each at 0.4 nM, 40 nM, and 400 nM.

Nanobodies directed to enzymes reportedly show a propensity to block enzymatic activity^25,26^. CD38 catalyzes the synthesis of cyclic ADP-ribose and ADP-ribose from NAD^+^ and the synthesis of cyclic GDP-ribose (cGDPR) from nicotinamide guanine dinucleotide (NGD^+^)^4^. Since the latter can be monitored conveniently by fluorimetry, we used this GDPR-cyclase assay to analyze the capacity of CD38-specific nanobodies to modulate the enzymatic activity of CD38. CD38-specific nanobodies from 22 families were analysed for their capacity to modulate the GDPR-cyclase activity of CD38 (Fig. 1). Three nanobodies (JK2, MU1067, MU523, families 4, 20, 19) inhibited the conversion of NGD^+^ to cGDPR by recombinant CD38 in a dose-dependent manner. Two other nanobodies (WF14 and MU738, families 7 and 9) enhanced CD38-catalyzed synthesis of cGDPR.

### Crossblockade analyses reveal binding of nanobodies to three non-overlapping epitopes

Next, we aimed to assess whether the selected nanobodies recognize overlapping or distinct epitopes on CD38. To this end, we performed crossblockade flow cytometry analyses with Alexa^647^-conjugated nanobodies from nine different families in the presence of excess unlabeled nanobodies (Table 2, Figure S5). The results allowed grouping of the selected nanobodies into three distinct non-overlapping bins. Group 1 nanobodies block binding of nanobodies MU1068 (family 12), WF211 (family 17), and MU274 (family 13). These nanobodies recognize an overlapping epitope designated epitope 1. Similarly, group 2 nanobodies recognize an epitope overlapping with the binding epitope of nanobodies JK2 (family 4), MU1067 (family 20), and MU523 (family 19), designated epitope 2. Of note, these three epitope-2 nanobodies are the most potent antagonists of the enzyme activity of CD38 (Fig. 1). Group 3 nanobodies block binding of nanobodies JK19 (family 15), JK36 (family 2) and WF100 (family 22) designated epitope 3.

**Table 2:**
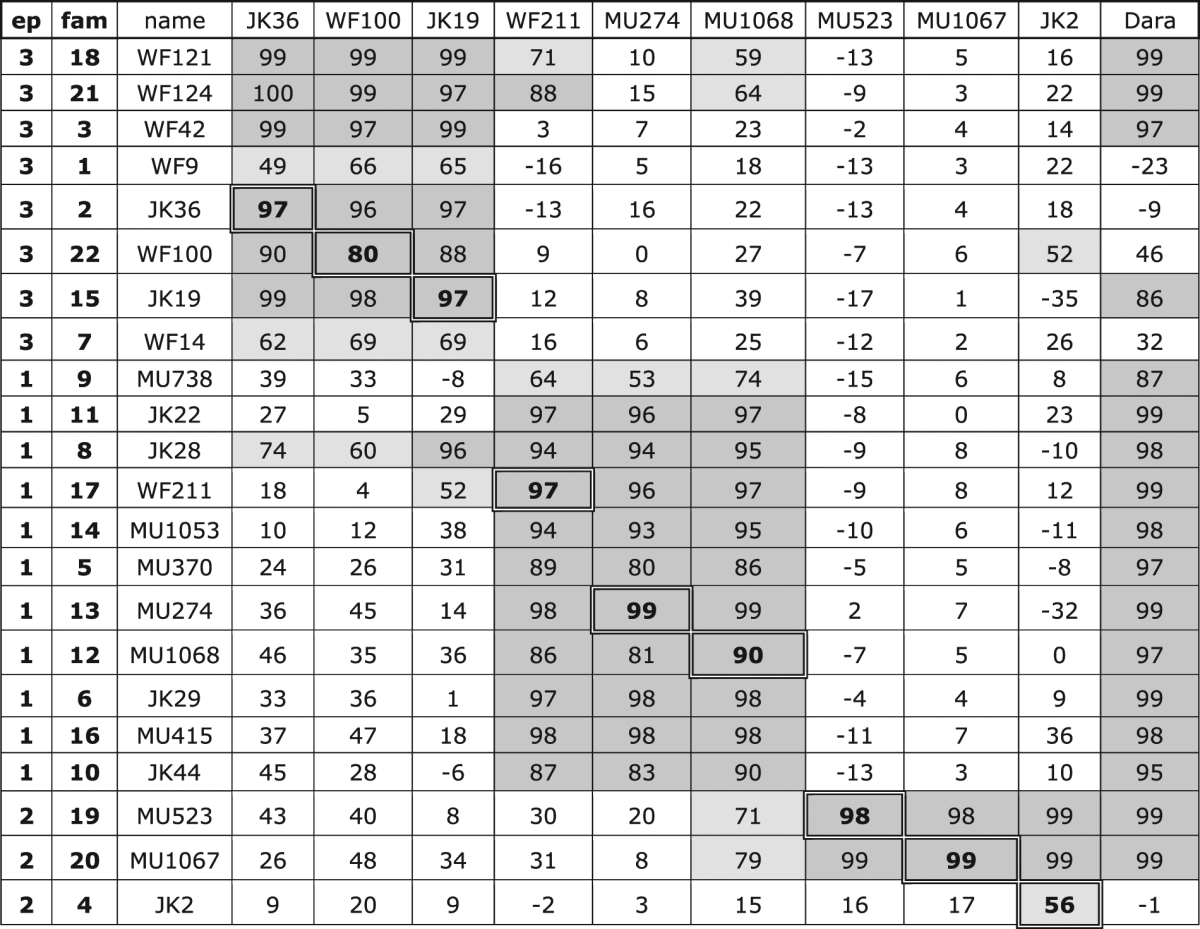
Epitope mapping of CD38-specific nanobodies.

CA46 lymphoma cells were preincubated for 30 min at 4 °C with unconjugated nanobodies (indicated on the left) before addition of Alexa^647^-conjugated nanobodies (indicated on top). Cells were further incubated for 30 min at 4 °C, washed and analyzed by flow cytometry. Numbers indicate the percentage maximal blockade of the mean fluorescence intensity of cells labeled in the absence of competing nanobodies, negative numbers indicate enhanced labeling of cells in the presence of the competing nanobody. Inhibition of binding by 50–80% is highlighted in light grey, inhibition of binding by >80% in dark grey. Self-blockade by the nanobody used for labeling is indicated by highlighted boxes in the diagonal.

### Four nanobody families bind CD38 independently of daratumumab

In order to perform comparative binding analyses with monovalent CD38 nanobodies, we cloned the antigen-binding domain of daratumumab in a monovalent scFv format, designated Dara scFv (Figure S6). We performed cross-blockade analyses with Alexa^647^ conjugated Dara scFv to determine which of the CD38-specific nanobodies could bind to CD38 independently of Dara scFv. Interestingly, binding of Dara scFv was blocked by preincubation of CD38-expressing cells with nanobodies from each of the three epitope groups, including all epitope 1 families (Table 2, Figure S5). Three nanobodies did not interfere with binding of Dara scFv: one out of three epitope group 2 (JK2, family 4) and two out of eight epitope group 3: WF9 and JK36, (families 1 and 2). Two additional epitope group 3 nanobodies, MU1105 and WF14 (families 7 and 22) partially blocked binding of Alexa^647^-Dara scFv.

**Figure 2:**
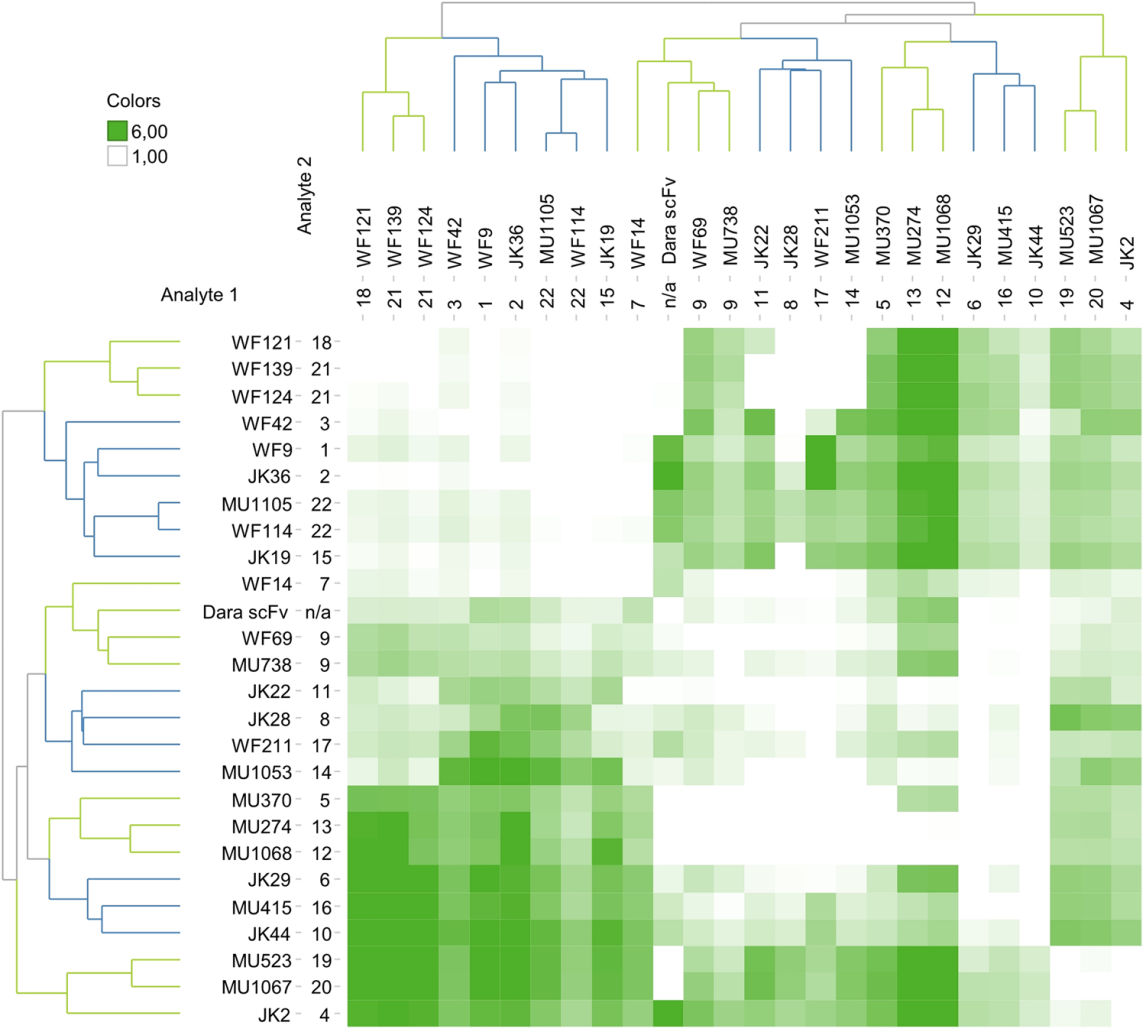
In-tandem binding analyses of nanobodies to immobilized recombinant CD38 by BLI. Sequential binding analyses of nanobodies to the glycosylated extracellular domain of CD38 immobilized on AR2 Biosensors were performed using the Octet RED384. Hierarchial clustering was performed using the Ward’s method. Y-axis refers to first loaded analyte, Y-axis to second analyte. White: no additional binding of the second agent was observed, e.g. epitope was occupied or hindered by the loaded agent. Green: additional binding of the second agent was observed. Self-binning is indicated along the diagonal in white boxes.

In addition, the complete panel of nanobodies from 22 families and Dara scFv was assessed for in-tandem binding by BLI analysis to allow further mapping of subgroups within the three main clusters. Nanobodies were non-conjugated and simultaneous binding was assessed in both orders, i.e. injection as first or second analyte (Fig. 2, Table S1). Within epitope group 1, several families competed with a selection of families within epitope group 3, which may indicate that these families recognize a bridging epitope located between 1 and 3. For most nanobodies binding was independent of the order of injection, but for some, like WF14 in family 7 and MU738 in family 9, the tandem binding profile was different dependent on the order. Interestingly, families 7 and 9 were identified as potentiators of CD38 enzyme activity (Fig. 1), and hence it is conceivable that these sensitize CD38 by stabilization of a more active conformation.

These results of in-tandem epitope binning analyses of Dara scFv with CD38-specific nanobodies confirmed the independent binding of Dara scFv and nanobody families 1, 2, and 4. Moreover, three distinct members of family 22 were shown to bind CD38 in conjunction with Dara scFv, irrespective of the order of injection, while binding of family 7 was only observed when Dara scFv was allowed to bind first, in support of a conformational mechanism (Figure S7, Table S1). Hence within epitope group 3, families 1, 2 and 22, and within epitope group 2, family 4, represent subgroups that bind to an epitope that is non-overlapping with Dara scFv. Taken together, CD38 nanobodies from 4 distinct families and two non overlapping epitope groups are capable of binding CD38 in conjunction with Dara scFv.

### Nanobodies bind to human CD38 on lymphoma cell lines, peripheral blood NK and B cells, and primary myeloma cells

**Figure 3:**
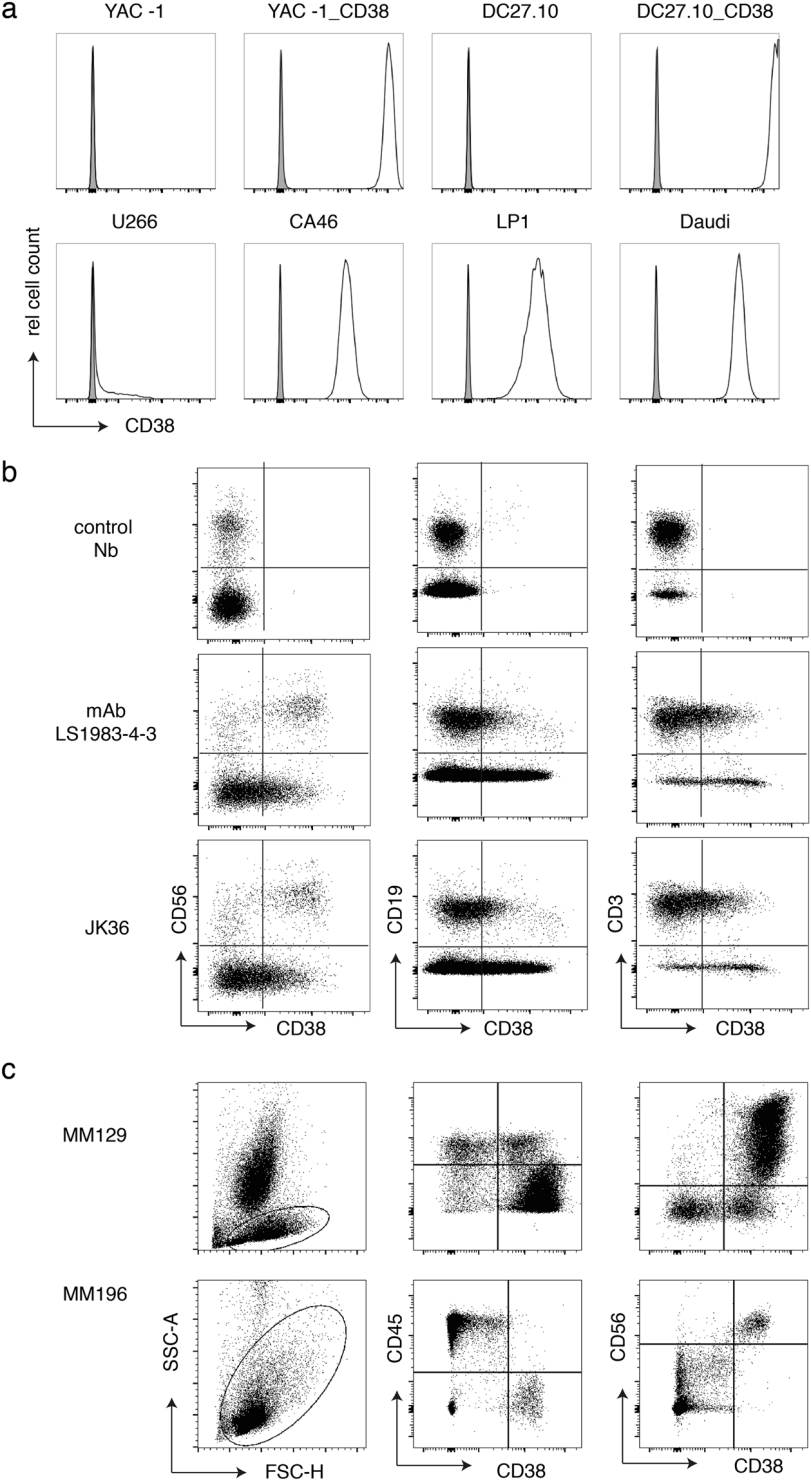
Fluorochrome-conjugated nanobodies detect CD38 on the surface of lymphoma cell lines, peripheral blood lymphocytes, and primary myeloma cells. (**a**) Untransfected mouse lymphoma cell lines Yac-1 and DC27.10 and their counterparts stably transfected with human CD38 (top row of panels) and human lymphoma cell lines (bottom row) were stained with Alexa^647^-conjugated nanobody MU1067 or an irrelevant control nanobody. (**b**) Blood samples from normal donors were incubated with fluorochrome-conjugated antibodies against CD45 (pan-lymphocytes), CD56 (NK cells), CD19 (B cells) and CD3 (T cells) and CD38-specific mAb LS1983-4-3, Nb JK36, or a control nanobody for 30 min at RT. Erythrocytes were lysed and cells were analyzed by flow cytometry. Gating was performed on CD45+ lymphocytes. (**c**) Bone marrow samples from two myeloma patients were incubated with fluorchrome-conjugated antibodies against CD45 and CD56 and nanobody MU1067 for 30 min at RT. Erythrocytes were lysed and cells were analyzed by flow cytometry. Gating was performed on lymphocytes. Myeloma cells in these patients express CD56 but do not express CD45.

Next, purified fluorochrome-conjugated monovalent anti-CD38 nanobodies were analyzed for binding to native CD38 on the cell surface of human tumor cells, NK cells and B cells (Fig. 3). The results confirm high level of CD38 expression by established tumor cell lines derived from multiple myeloma (LP-1) and Burkitt’s lymphoma (CA46, Daudi) (Fig. 3a). On peripheral blood leukocytes of normal donors, all nanobodies showed high level staining of CD16^+^ NK cells and a subset of CD19^int^ B cells and much lower staining of T cells and CD19^hi^ B cells, consistent with the known expression of CD38 by these cells (Fig. 3b). The same staining pattern was observed with the conventional mAb LS198–4–3 that is commonly used for routine diagnostics. We further analyzed the utility of the nanobodies to detect tumor cells in primary bone marrow samples from patients with multiple myeloma. The results show specific discrimination of myeloma cells (CD45^lo^/CD56^hi^) with CD38-specific nanobodies (Fig. 3c).

**Figure 4:**
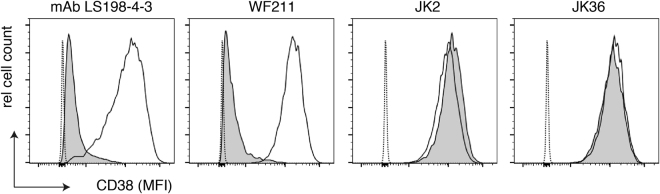
Fluorochrome-conjugated nanobodies JK2 and JK36 detect CD38 on the surface of human lymphoma cell lines saturated with daratumumab. LP-1 myeloma cells were preincubated with a saturating dose of DarzalexR, (daratumumab, human IgG1) (grey histograms) or with PBS (open histograms) before addition of fluorochrome-conjugated CD38-specific mAb LS198-4-3 or monovalent nanobodies WF211, JK2, or JK36. After incubation for 20 min at 4 °C cells were washed and analyzed by flow cytometry. Dashed lines = unstained cells.

We next set out to determine whether the nanobodies that bind independently of Dara scFv to a non-overlapping epitope could also stain tumor cells in a therapeutic setting, i.e. when saturated with intact daratumumab. To this end, we preincubated LP-1 myeloma cells with a large excess of Darzalex® before incubation with fluorochrome conjugated nanobodies (Fig. 4). The results show that nanobodies JK2 and JK36 effectively stain cell surface CD38 even after opsonization with daratumumab.

### Specific detection of CD38^+^ tumors *in vivo* with nanobody MU1067 conjugated to the near infrared dye Alexa^680^

**Figure 5:**
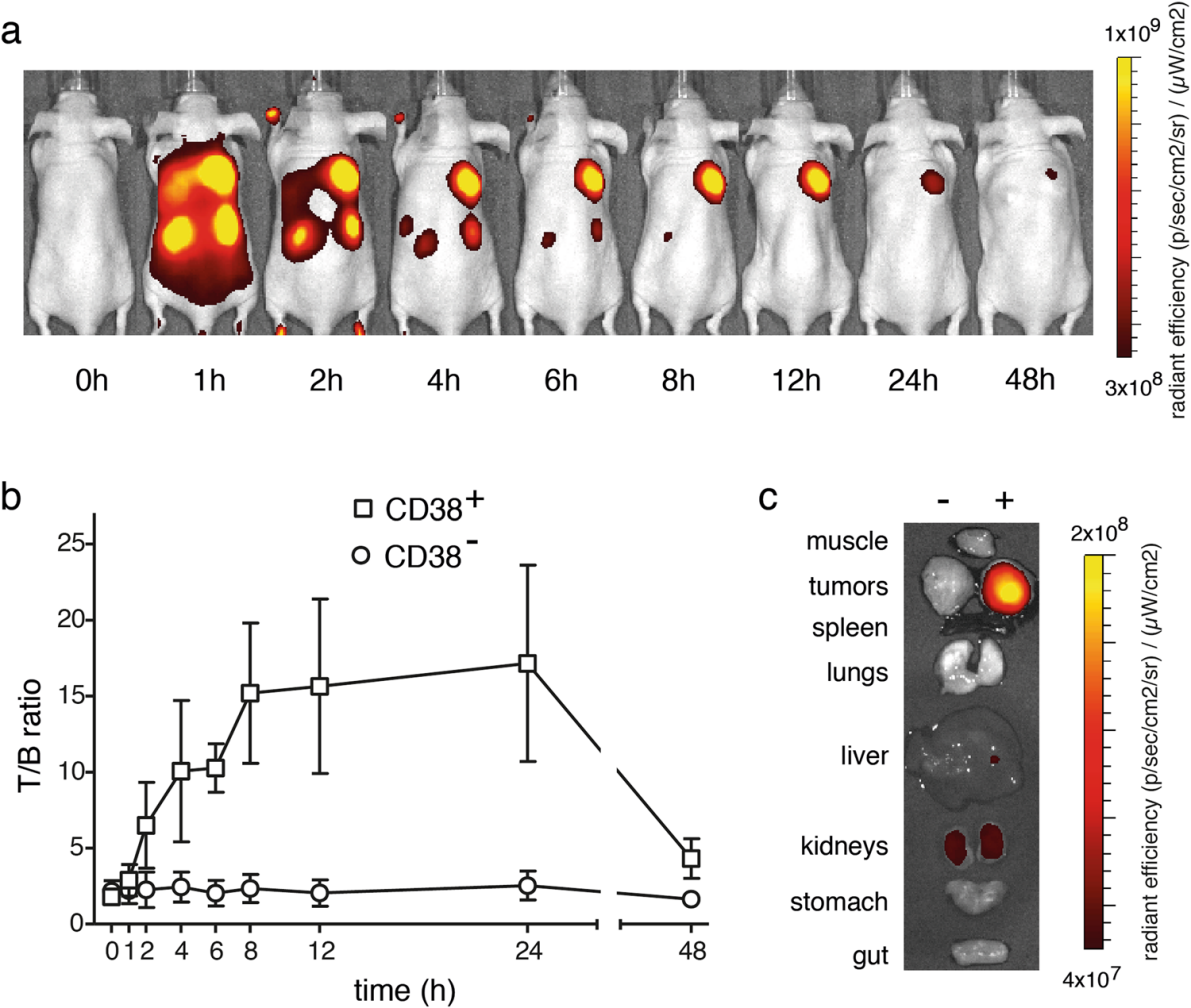
Alexa^680^-conjugated nanobodies provide excellent discrimination of CD38^+^ tumors *in vivo*. Untransfected DC27.10 mouse lymphoma cells and DC27.10 cells stably transfected with human CD38 were injected subcutaneously in the left and right flanks of Nude mice. Seven days after tumor implantation, 50 µg of Alexa-680-conjugated nanobody MU1067 was injected into the tail vein. (**a**) Optical molecular imaging was performed before and at the indicated time points after nanobody injection. Signals below the tumors correspond to the kidneys. Signals on the tail and feet likely reflect contact with urine containing labeled nanobodies. (**b**) T/B ratios were determined from regions of interests (ROIs) drawn around tumors and normal tissue (hind leg) for semi-quantitative analyses. T/B ratios were calculated by subtracting background signals from radiant efficiencies of ROIs around tumors. T/B ratios are plotted as a function of time. (**c**) Optical molecular imaging of tissues removed after sacrifice 48 h after nanobody injections.

Next we determined whether CD38-specific nanobodies could be used as imaging agents to detect CD38 expressing tumors *in vivo* (Fig. 5). To this end we used a two-sided tumor model in nude mice bearing untransfected and CD38-transfected lymphoma cells injected subcutaneously in the left and right flanks. In order to allow *in vivo* imaging with the IVIS200 system, nanobody MU1067 was conjugated to the near infrared dye ALexa^680^ and specific binding of Alexa^680^-MU1067 to CD38-expressing cells was confirmed by flow cytometry. Seven days after injection of tumor cells, Alexa^680^-MU1067 (50 µg/mouse, 2.5 mg/kg) specifically detected CD38^+^ tumors *in vivo* already within 1 hour after nanobody injection (Fig. 5a,b). At this time point very strong signals were also detected in the kidneys, consistent with renal excretion of excess unbound nanobody (15 kD). At 2 hours after injection, signals from the CD38^+^ tumor exceeded those of the kidneys. At the time of sacrifice (48 h post injection) the CD38^+^ tumors continued to show high signals, while signals in other tissues returned to background levels (Fig. 5c), with low fluorescent signals still detectable in kidneys. While the liver itself showed only background fluorescence, fluorescent signals in the gall bladder at the time of sacrifice likely reflect biliary excretion of fluorochromes. In conclusion, in the time window from 2–24 h post injection, high tumor/background ratios were observed in all animals (Fig. 5b).

## Discussion

The goal of this study was to generate nanobodies directed against the cell surface ecto-enzyme CD38 as new diagnostic and potential therapeutic tools for hematological malignancies. We successfully identified 22 families of CD38-specific nanobodies from phage display libraries generated from immunized llamas. Our results show that some of these nanobodies modulate the enzymatic activity of CD38 and allow specific detection of CD38 expressing tumors *in vivo*.

11 of 22 nanobody families were obtained from protein-immunized llamas after panning on the aglycosylated CD38 ectodomain, the other 11 nanobody families were obtained from cDNA-immunized llamas by binding to CD38-transfected cells in solution. Since the four llamas used were outbred and genetically diverse, it is not possible to conclude that one or the other strategy is better. However, it is perhaps noteworthy that three of four families that bind CD38 independently of daratumumab (families 1, 2, and 4) were derived from genetic immunizations whereas the clone with the highest affinity (MU523, family 19) was derived from a llama immunized with protein.

**Figure 6:**
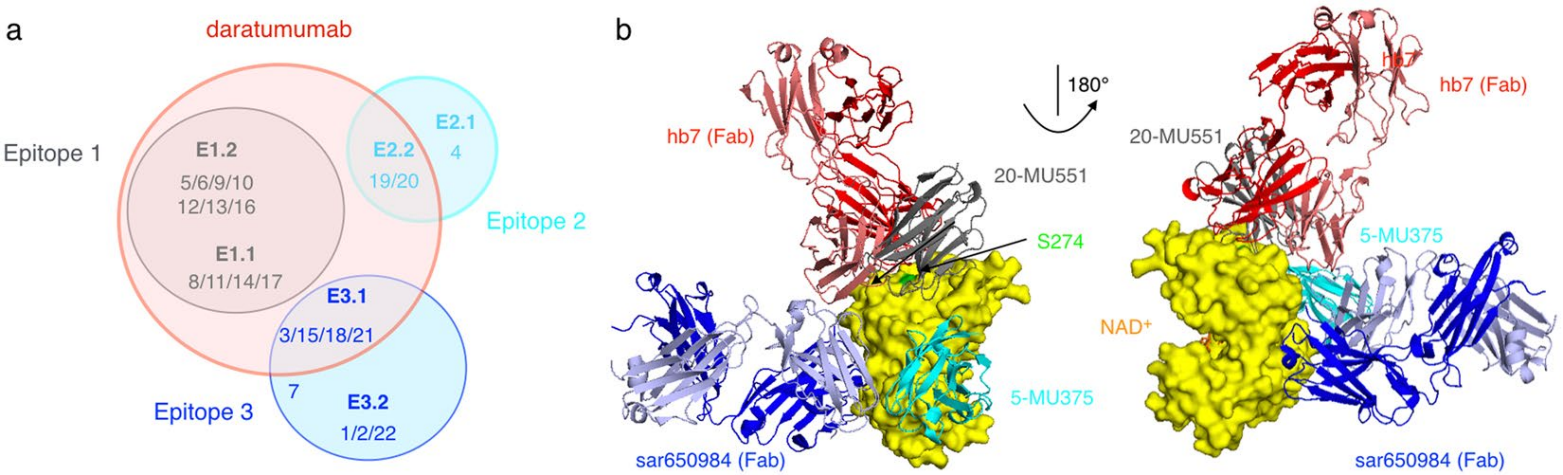
Venn diagram of binding epitopes and structure models of CD38 in complex with nanobodies and mAbs. (**a**) Venn diagram of binding epitopes on CD38 for nanobodies and the single chain variable fragment of daratumumab. Numbers indicate family numbers of CD38-specific nanobodies. (**b**) 3D-structures of CD38 (yellow) in complex with NAD^+^ (orange), nanobodies MU375 (cyan, family 5), MU551 (grey, family 20), or the Fab fragment of mAb hb7 (red) or of mAb sar650984 (blue) were aligned with PyMOL using PDB-IDs 2i65, 5f21, 5f10, 3raj, and 4cmh, respectively. Mutation of serine 274 (green) to phenylalanine abolishes binding of daratumumab.

All CD38 nanobodies bind to three independent non-overlapping epitopes (Fig. 6a). Interestingly, all nanobodies from epitope binning group 1 and many nanobodies from epitope groups 2 and 3 interfered with binding of Dara scFv. The nanobody CDR3 loop can fold over a side of the variable domain to increase the interaction surface with the antigen and the solvent accessible surface area of a nanobody can be as large as that of a VH-VL pair^25^. However, the size of the pair of variable domains of Dara scFv is roughly twice as large as that of a the single variable domain of a nanobody. Consistently, the results of the tandem epitope binning analyses by Octet show that the binding site of Dara scFv is larger than that of the CD38-specific nanobodies. Although there is no structural data available on the binding of Daratumumab, it presumably uses both its VH and VL domains for binding to CD38, i.e. it can be expected to cover roughly twice as large a surface area of CD38 than the nanobodies. One nanobody family of epitope group 1 (JK2, family 4) and a subgroup of three nanobody families within epitope group 3 (WF9, JK36, and MU1105, families 1, 2 and 22) bound CD38 independently of daratumumab. Nanobodies JK2 and JK36 effectively recognize cell surface CD38 even after opsonization with saturating doses of daratumumab. These nanobodies could potentially be used to monitor expression of CD38 on the cell surface of lymphocytes and tumor cells in daratumumab-treated patients.

We have previously determined the precise epitopes of three different CD38 nanobodies within epitope groups 1 and 2 by co-crystallisation with the CD38 ectodomain (Fig. 6b). Structural information for nanobodies MU375 (fam 5, epitope 1), MU1053 (fam 14, epitope 1), and MU551 (fam 20, epitope 2) is available in PDB codes 5F21, 5F1O and 5F1K, respectively^21^. All three nanobodies compete with the single chain variable fragment of daratumumab for binding to cells and to recombinant protein, suggesting overlapping epitopes. In line with this, Ser274 of CD38 (Fig. 6b), which is described to be important for daratumumab binding^13^, is part of the footprint on CD38 in each of the two epitope 1 nanobodies^21^. Within epitope group 1, family 14 differs from family 5 in that it competes with a subset of epitope group 3 nanobodies. The structural data confirms that MU1053 (fam14) is more directed to the hind site of the protein, further away from the catalytic pocket than MU375 (fam5).

Previous studies have uncovered a striking propensity of nanobodies from immunized llamas to bind to the active site of enzyme antigens^20,23,25–27^. In our study, three of 22 nanobody families, i.e. all epitope 2 nanobodies, blocked CD38-catalyzed conversion of NGD^+^ to cyclic GDPR in a dose dependent fashion, whereas two nanobody families (families 7 and 9 from epitope groups 3 and 1, respectively) potentiated CD38 enzyme activity. In this context it is also of interest to note that only one of well over 100 monoclonal antibodies generated against CD38 has been shown to inhibit the enzyme activity of CD38^28^. The crystal structure of this mAb sar650984 in complex with CD38 (Fig. 6b) revealed binding of sar650984 far away from the active site crevice (Fig. 6b), implying an allosteric mode of action^28^. It seems likely that the enzyme-inhibiting nanobodies similarly act in an allosteric fashion, considering that the binding site of the MU551 family 20 nanobody is also located away from the active site crevice (Fig. 6b). Similarly, the nanobodies that were found to sensitize the catalytic activity of CD38 may act in an allosteric manner, given the observation of conformational constraints for these nanobodies in the tandem binding studies. It has been suggested that metabolites of NAD^+^ generated by CD38 in the tumor microenvironment can promote tumor growth and immunosuppression^8^. Thus, it is conceivable that blocking the enzymatic activity of CD38 may be of therapeutic benefit in cancer. If so, this could influence the choice of nanobodies as therapy candidates for pre investigational new drug experiments. In particular, use of the antagonistic nanobody family 4 that binds independently of daratumumab, would be feasible even in patients undergoing daratumumab treatment. It will thus be interesting to determine whether allosteric modulation of CD38-enzyme activity *in vivo* by CD38 nanobodies can counteract its purported immunosuppressive and tumor promoting effects in the tumor microenvironment^8^.

In a subcutaneous xenograft tumor model in nude mice, we examined the capacity of CD38-specific nanobodies to specifically target CD38-expressing tumor cells. In this model, the subcutaneous location and the nude skin minimized quenching of fluorescent signals from the NIRF-conjuagted nannobodies by muscle, bone or hair and thus facilitated *in vivo* imaging. Moreover, since the nanobodies do not cross react with mouse CD38, the mouse model provided a clear background. The results of these experiments clearly demonstrate the capacity of nanobodies to specifically target CD38^+^ vs. CD38^−^ tumors. In a clinical setting, radionuclide-labeled nanobodies can be expected to provide higher sensitivity at lower doses^29^, but higher background signals due to binding of nanobodies to endogenously expressed CD38 in healthy tissues. Besides the high specificity and affinity to CD38, the efficient imaging of CD38^+^ tumors can be attributed to the small size of the nanobody which allows excellent tissue and tumor penetration^15,16,29^ and fast clearance of excess unbound nanobodies from the circulation by renal excretion^30^. Hence the current panel of high affine monovalent CD38-specific nanobodies are attractive for use as companion diagnostic for anti-CD38 therapies.

For therapeutic applications, nanobodies can readily be humanized, e.g. by fusion to the hinge and Fc-domains of human IgG1^31^. Moreover, the VHH domain itself can be humanized by substituting framework residues to more closely resemble those of human VH domains^32^. This is done routinely for llama-derived nanobodies in clinical development^33,34^.

In conclusion, our results underscore the potential of nanobodies for modulating the enzymatic activity of CD38 and for specific *in vivo* detection of CD38^+^ tumors. Importantly, we describe four nanobody families that bind independently of daratumumab, which could potentially be valuable for monitoring the efficacy of daratumumab therapy since they can still detect CD38 after binding of daratumumab. The nanobodies reported here thus hold promise as new diagnostic and potential therapeutic tools for multiple myeloma and other CD38-expressing malignancies.

## Methods

### Protein production and llama immunizations

The extracellular domain (aa 46–300) of a variant of CD38 in which the three potential N-linked glycosylation sites were inactivated was produced as a secretory protein in yeast cells and purified as described previously^3^. The extracellular domain of CD38 (aa46–300) with intact glycosylation sites was produced as a secretory protein with a chimeric His6x-Myc epitope tag in the pCSE2.5 vector^22^ (kindly provided by Dr. Thomas Schirrmann, Braunschweig). For cDNA immunization the full-length open reading frame of CD38 was cloned into the pEF-DEST51 expression vector. Two llamas (*Lama glama*) (designated 10, 25) were immunized subcutaneously with purified recombinant aglycosylated protein emulsified with Specol adjuvant (240 µg in 500 µl total volume)^20,27,35^. Two llamas (designated 538 and 539) were immunized by ballistic cDNA immunization^20,36^. The humoral immune response was monitored in serially diluted serum by ELISA on microtiter plates (Nunc MaxiSorp, Thermo Fisher Scientific, Waltham, MA) coated with recombinant CD38, using monoclonal antibodies directed against llama IgG2 and IgG3 kindly provided by Dr. Judith Appelton, Cornell University, NY^37^. Animals were bled 4–18 days after the 3rd or 4th boost.

### Cells

The Yac-1 and DC27.10 mouse lymphoma cell lines were transfected with linearized full-length human CD38 expression vector pEF-DEST51. Stable transfectants were selected in medium containing blasticidin and by fluorescence activated cell sorting. Human multiple myeloma (RPMI-8266, U266, LP-1) and Burkitt’s lymphoma (CA46, DAUDI) cell lines were obtained from the Leibniz-Institute DSMZ-German Collection of Microorganisms and Cell Cultures, Braunschweig, Germany. Bone marrow aspirates of patients MM123 and MM129 were obtained after written informed consent as approved by the ethics committee (Ethikkommission der Ärztekammer Hamburg, PV4767).

### Construction of phage display library and selection of CD38-specific nanobodies

Mononuclear cells were isolated from 120 ml of blood by Ficoll-Paque^TM^ (GE Healthcare, Chalfont St Giles, UK) gradient centrifugation. RNA purified from these cells by TRIZOL reagent (Invitrogen, Carlsbad, CA) was subjected to cDNA synthesis with random hexamer primers. The VHH coding region was amplified by PCR with degenerate VHH-specific primers^20,23^. PCR products were purified from agarose gels, digested sequentially with SfiI and NotI (NEB, Ipswich, MA) and cloned into the pHEN2 phagemid vector downstream of the PelB-leader peptide and upstream of the chimeric His6x-Myc epitope tag^27,38^. Transformation into XL1-Blue *E. coli* (Stratagene, La Jolla, CA) yielded libraries with sizes of 4.0 × 10^5^–10^7^ clones. Phage particles were precipitated with polyethylene glycol from culture supernatants of *E. coli* transformants infected with a 10-fold excess of M13K07 helper phage (GE Healthcare, Chalfont St Giles, UK).

Panning of specific phage was performed using either the recombinant aglycosylated human CD38 ectodomain immobilized on microtiter plates (Nunc MaxiSorp, Thermo Fisher Scientific, Waltham, MA) or in solution with CD38-transfected Yac-1 cells. Phage particles (1.6 × 10^11^) were incubated with recombinant CD38 or CD38-transfected cells for 60 min with agitation at room temperature in PBS, 10% Carnation non-fat dry milk powder (Nestlé, Glendale, CA). Following extensive washing, bound phages were eluted from ELISA plates with 50 mM diethylamine and neutralized with 1 M Tris-HCl pH 8. Phages were eluted from transfected cells by trypsinization. Eluted phages were titrated and subjected to one or two more rounds of panning, following the same procedure. Phage titers were determined at all steps by infection of TG1 *E. coli* cells (Stratagene, La Jolla, CA). Plasmid DNA was isolated from single colonies and subjected to sequence analyses using pHEN2-specific forward and reverse primers.

### Production and reformatting of nanobodies

Monomeric nanobodies were expressed in HB2151 *E. coli* cells (GE Healthcare, Chalfont St Giles, UK)^20,23^. Protein expression was induced with IPTG (Roche, Rotkreuz, Switzerland) when bacterial cultures had reached an OD_600_ of 0.5 and cells were harvested after further cultivation for 3–4 h at 37 °C. Periplasmic lysates were generated by osmotic shock and removal of bacterial debris by high speed centrifugation. Nanobodies were readily purified from *E. coli* periplasmic lysates by immobilized metal affinity chromatography (IMAC).

The coding region of selected nanobodies was subcloned using NcoI/PciI and NotI upstream of a chimeric His6x-Myc epitope tag into the pCSE2.5 vector^22^ (kindly provided by Thomas Schirrmann, Braunschweig). Daratumumab scFv was generated by gene synthesis using the published sequence (WO 2011/154453) by fusing the VH domain and the VL domain via a 15GS linker flanked by NcoI and NotI sites and cloned upstream of a chimeric His6x-Myc epitope tag into the pCSE2.5 vector.

Recombinant myc-his tagged nanobodies and Dara scFv were expressed in transiently transfected HEK-6E cells cultivated in serum-free medium^23,36^. Six days post transfection, supernatants were harvested and cleared by centrifugation. Nanobodies in cell supernatants were quantified by SDS-PAGE and Coomassie staining relative to marker proteins of known quantities: 10 µl samples of the supernatant were size fractionated side by side with standard proteins (albumin 4 µg, IgH 2 µg, IgL 1 µg, lysozyme 0.4 µg; albumin 1 µg, IgH 0.5 µg, IgL 0.25 µg, lysozyme 0.1 µg). Yields of recombinant nanobodies typically ranged from 0.5–3 µg/10 µl. Myc-His tagged nanobodies were purified by immobilized metal affinity chromatography using Ni-NTA agarose (Sigma, St Louis, MO).

### ELISA

Recombinant CD38 (100 ng/100 µl PBS/well) was adsorbed to 96-well Nunc MaxiSorp plates (Thermo Fisher Scientific, Waltham, MA) at 4 °C over night. Wells were washed twice with PBS and blocked for 2 hours with PBS containing 5% nonfat powdered milk at room temperature. Wells were incubated for 30 min with llama pre and immune serum (diluted 1:100 in PBS). Following washing with PBS/0.05% Tween 20, bound antibodies were detected with llama IgG-specific mAbs followed by peroxidase-conjugated anti-mouse IgG (Jackson) and (TMB) (Sigma, St Louis, MO) as substrate. The absorbance at 450 nm was measured using a Victor3 ELISA-reader (Perkin-Elmer, Waltham, MA).

### Off-rate determination

Off-rates of CD38 nanobodies were determined by BLI technology, using an Octet RED384 instrument (ForteBio). As running buffer HBS-EP + (0.01 M HEPES pH 7.4, 0.15 M NaCl, 3 mM EDTA, 0.05% v/v Surfactant P20) was used. Assays were performed at 25 °C. The shake speed during the biosensor preparation and off-rate determination was set at 1000 rpm. Amine reactive 2^nd^ Generation (AR2G) biosensors (ForteBio) were activated for 10 minutes with EDC(20 mM)/NHS(10 mM) and recombinant human CD38 was loaded at 10 µg/ml in 10 mM sodium acetate pH 6 for 15 min. After immobilization, surfaces were deactivated with 1 M ethanolamine (pH 8.5) for 10 min. During off-rate screening 100 nM and 1 µM nanobody were allowed to associate during 5 min on immobilized human CD38 followed by a 10 min dissociation. After each cycle the human CD38 surfaces were regenerated via 5 short pulses of 5 s of 100 mM HCl and running buffer. Data processing and off-rate determination was performed with ForteBio Data Analysis Software Version 9.0.0.12. Sensorgrams were double referenced by subtracting 1) running buffer on reference biosensor containing only human CD38 and 2) nanobody interaction on parallel reference biosensors on which no human CD38 was immobilized. Processed curves were evaluated via a fitting with the model ‘1:1’.

### CD38 epitope binning

In-tandem epitope binning of CD38-specific nanobodies and Dara scFv was performed on an Octet RED384 instrument (ForteBio). As running buffer HBS-EP + (0.01 M HEPES pH 7.4, 0.15 M NaCl, 3 mM EDTA, 0.05% v/v Surfactant P20) was used. Experiments were performed at 20 °C. The shake speed during the biosensor preparation and epitope binning was set at 1000 rpm. Amine reactive 2^nd^ Generation (AR2G) biosensors (ForteBio) were activated for 10 min with EDC(20 mM)/NHS(10 mM) and human CD38 protein was loaded at 10 µg/ml in 10 mM sodium acetate pH6 for 15 min. After immobilization, surfaces were deactivated with 1 M ethanolamine (pH 8.5) for 10 min. In the epitope binning, 100 nM nanobody 1 was loaded during 3 min on immobilized CD38 to saturate all available epitopes. Nanobody 2 was presented after a 10 s dip in running buffer for 3 min followed by a 1 min dissociation. After each cycle the human CD38 surfaces were regenerated via 5 short pulses of 5 s each of 100 mM HCl followed by running buffer. Data was processed with ForteBio Data Analysis Software Version 9.0.0.12. Binding levels of nanobody 2 were determined at the end of the 3 min association and compared to levels at baseline (beginning of association). Irrelevant nanobody controls were included. The binding level of nanobody 2 for each nanobody 2 - nanobody 1 pair was divided by the binding response of nanobody 2 on a CD38 surface saturated with nanobody 2 (self-binning). Normalized data was hierarchically clustered using Ward’s method (distance measure: half square Euclidian distance; scale: logarithmic) and visualized in Spotfire (TIBCO Software Inc.).

### Fluorimetric enzyme assay

CD38 catalyzes both, the synthesis of cADPR and nicotinamide from β-NAD^+^, and the fast hydrolysis of cADPR to ADPR. A fluorimetric enzyme assay with slower kinetics has been developed using nicotinamide guanine dinucleotide (NGD^+^) as substrate^4^. NGD^+^ is converted to cyclic GDP-ribose (cGDPR) and nicotinamide followed by a very slow hydrolysis of cGDPR to GDPR, leading to accumulation of the fluorescent product cGDPR. Enzymatic production of cGDPR from NGD^+^ (80 µM, Sigma, St Louis, MO) was monitored continuously for 50 min at 410 nm (emission wavelength) with an excitation wavelength set at 300 nm, using a Hitachi F-2000 fluorimeter. Anti-CD38 nanobodies were pre-incubated at a final concentration of 400, 40, 4, and 0.4 nM with 5 nM recombinant glycosylated extracelluar domain of CD38 for 15 min at RT before addition of NGD^+^ and further incubation in the dark at RT in triplicate wells for each treatment. Readings (EX300/EM410) from wells without CD38 were subtracted from all sample readings and were plotted for each nanobody concentration in Relative Fluorescence Units (RFU) *vs*. time. The rate of cGDPR production was calculated as the slope of these curves (RFU/s) during the linear phase of the reaction, i.e. between t = 10 min and t = 20 min.

### Flow cytometry

Untransfected Yac-1 cells and Yac-1 cells stably transfected with human CD38 were incubated for 30 min with nanobody-containing periplasmic lysates (diluted 1:10 in PBS). Following washing with PBS/0.1% BSA, bound nanobodies were detected with FITC-conjugated anti-c-Myc mAb 9E10 (Sigma, St Louis, MO). Human tumor cell lines, peripheral blood leukocytes, and bone marrow cells were incubated for 30 min with fluorochrome-conjugated nanobodies and monoclonal antibodies directed against CD3 (SK7), CD16 (3G8), CD19 (HIB19), CD45 (HI30), and CD38 (LS198–4–3) (BD Biosicences, Heidelberg and Beckman-Coulter, Krefeld). Darzalex® was purchased from Janssen-Biologics, Leiden. For binding stability analyses, CD38-transfected cells were incubated for 60 min with serial dilutions of monovalent nanobodies in PBS/0.1% BSA. Cells were washed three times with PBS/0.1% BSA. Bound antibodies were detected with FITC-conjugated anti-c-Myc mAb 9E10 (BioLegend).

For nanobody dissociation analyses, two separate aliquots of CD38-transfected cells were incubated either with Cell Proliferation Dye eFluor 450 (eBioscience) or with Alexa^647^-conjugated nanobodies for 20 min at 4 °C. Cells were washed four times, mixed at a 1:1 ratio and further incubated at 4 °C or at 37 °C for 0.5, 2, or 16 h before FACS analyses. The dissociation of nanobodies from the target cells and association with the eFluor 450 labeled cells was analyzed using the FlowJo software (Treestar).

### Cross-blockade analyses

For epitope analyses, cells were preincubated with excess monovalent nanobodies or Dara scFv (2 µg/100 µl PBS/0.1% BSA) for 30 min at RT before addition of fluorochrome-conjugated nanobodies (500 ng in 0.5 µl PBS) and further incubation for 20 min at RT. Cells were washed and analyzed by flow cytometry on a BD-FACS Canto. Data were analyzed using the FlowJo software (Treestar).

### *In vivo* and *ex vivo* imaging

Tumor graft experiments were conducted using athymic nude mice (NMRI-Foxn1nu) obtained from Charles River Laboratories (Sulzfeld, Germany). Experiments were performed in accordance with international guidelines on the ethical use of animals and were approved by the animal welfare commission (Amt für Verbraucherschutz, Lebensmittelsicherheit und Veterinärwesen Hamburg, Nr. 17/13). Prior to optical molecular imaging *in vivo*, 8–10-week-old mice were kept on an alfalfa-free diet for 7 d to minimize autofluorescence of the intestine. For generation of tumor grafts, mice were injected *s.c*. on the right side with 1 × 10^6^ CD38-transfected DC.27.10 cells and on the left side with 1 × 10^6^ untransfected DC27.10 cells, each in 0.2 ml of a 50:50 mix of RPMI medium and Matrigel (BD Biosciences, Franklin Lakes, USA). After 7 d, i.e. when tumors reached ~8 mm in diameter, 50 µg of Alexa^647^-conjugated nanobody MU1067 was injected *i.v*. via the tail vein. Similar doses have been found to yield good tumor to background ratios in previous studies using nanobodies conjugated to near infrared fluorochromes^30,39^. Optical molecular imaging was performed before injection and at indicated time points after injection. For optical molecular imaging, mice were anesthetized with isofluorane and positioned in the imaging chamber of a small animal imaging system (IVIS-200, Caliper Life Sciences, Hopkinton, Massachusetts, USA). After qualitative imaging *in vivo*, quantitative analyses were performed by placing ROIs around the CD38-positive tumors, the CD38-negative tumors (negative control) and the hind limb (background signal). Total radiant efficiency was determined with Living Image 4.2 software (Caliper Life Sciences). The tumor-to-background ratio was calculated by dividing the tumor uptake value by the background value. For *ex vivo* validation of *in vivo* measurements, animals were sacrificed 48 h post-injection. Tumors and organs (spleen, lungs, liver, kidneys, stomach, ileum, and muscle) were dissected and imaged with the IVIS-200.

## Electronic supplementary material

Supplementary Information
