# Supplementary material for: Nanobodies effectively modulate the enzymatic activity of CD38 and allow specific imaging of CD38+ tumors in mouse models in vivo
Source: Sci Rep. 2017 Oct 30;7:14289. doi: 10.1038/s41598-017-14112-6 (PMC5662768; doi:10.1038/s41598-017-14112-6)

Nanobodies effectively modulate the enzymatic activity of CD38 and allow specific imaging of CD38$^+$ tumors in mouse models *in vivo*

William Fumey[1,2], Julia Koenigsdorf[1,2], Valentin Kunick[1,2], Stephan Menzel[1], Kerstin Schütze[1,2], Mandy Unger[1], Levin Schriewer[1,2], Friedrich Haag[1], Gerhard Adam[2], Anna Oberle[3], Mascha Binder[3], Ralf Fliegert[4], Andreas Guse[4], Yong-Juan Zhao[5], Hon Cheung-Lee[5], Fabio Malavasi[6], Fernando Goldbaum[7], Rob van Hegelsom[8], Catelijne Stortelers[8], Peter Bannas[2], Friedrich Koch-Nolte[1]

[1]Institute of Immunology, [2]Department of Radiology, [3]Department of Oncology and Hematology, [4]Department of Biochemistry and Molecular Cell Biology, University Medical Center Hamburg-Eppendorf, D-20246 Hamburg, Germany

[5]School of Chemical Biology and Biotechnology, Peking University Shenzhen Graduate School (PKUSZ), 518052 Shenzhen, China

[6]Lab of Immunogenetics, Department of Medical Sciences, University of Torino School of Medicine, I-10126 Torino, Italy

[7]Fundacion Instituto Leloir, C1405 Buenos Aires, Argentina

[8]Albynx nv, B-9052 Zwijnaarde, Belgium

*correspondence and requests for materials should be addressed to F.K.-N. (nolte@uke.de) or P.B. (p.bannas@uke.de)

Supplementary information

Supplementary Figure S1. Llama immunizations with recombinant CD38 and CD38-encoding cDNA. a) Schematic diagram of the structure of CD38. The structure model is based on the crystal structure of the non-glycosylated extracellular domain of CD38 in complex with NAD (pdb code). CD38 is composed of an N-terminal intracellular domain (ic), a transmembrane domain (tm) and the extracellular C-terminal catalytic domain (green). The latter contains four N-linked glycosylation sites and six intrachain disulfide bonds (numbered 1-6). **b**) Two llamas (#10, #25) were immunized by intramuscular protein injection of the extracellular domain (aa 46-300) of a variant of CD38 in which the three potential N-linked glycosylation sites were inactivated. These llamas received four injections of CD38 (240 µg in 500 µl saline, emulsified in Specol adjuvant) at the indicated time points. Two llamas (#538, #539) were immunized by ballistic cDNA immunization on the shaved skin with a eukaryotic expression vector encoding full length human CD38. These llamas received four immunizations with CD38-encoding plasmid conjugated to 1 µm gold particles using a gene gun with a pressure setting of 600 psi (12 shots with 1 µg plasmid/mg gold particles per immunization). These llamas received a final boost with recombinant, glycosylated extracellular domain of CD38 (aa 46-300) C-terminally fused to a His6x-Myc tag (100 µg in 500 µl saline, emulsified in Specol adjuvant). Serum was prepared parallel to the isolation of peripheral blood lymphocytes at d8 - d10 after the final boost. Induction of a specific antibody response was verified by ELISA using immobilized recombinant CD38, yielding the following titers: 1:800 (#10), 1:1.600 (#25), 1:400 (#538) and 1:200 (#539). cDNA was prepared from blood lymphocytes obtained at 4-18 days after boost immunizations and used for generation of VHH-phage display libraries.

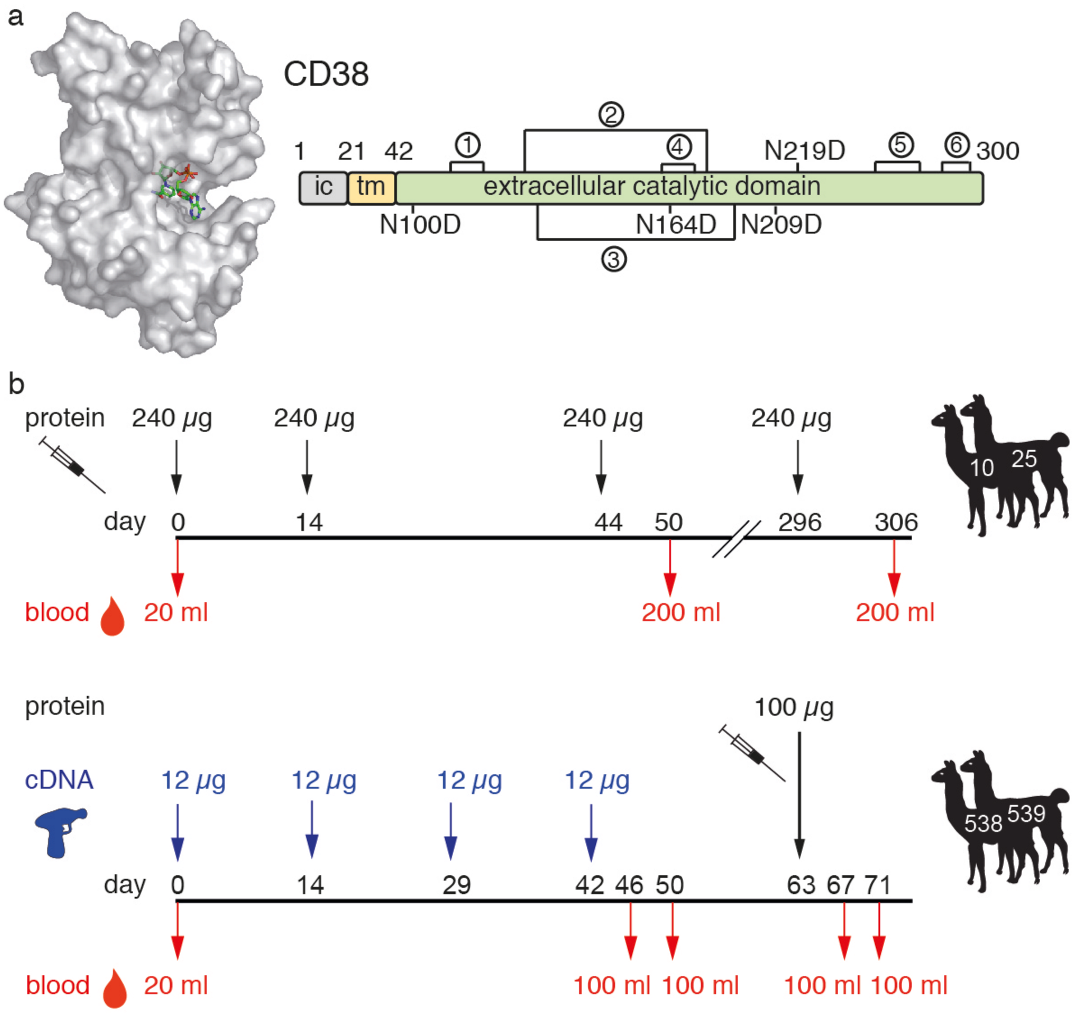

Supplementary Figure S2 | Selection of CD38-specific nanobodies. (**a**) A 1:1 mixture of untransfected and CD38-transfected DC27.10 cells was incubated for 20 min with periplasmic lysate. Cells were washed and bound nanobodies were detected with FITC-labeled anti-c-myc mAb (9E10). (**b**) Nanobodies showing the highest mean fluorescent intensity (MFI) staining of CD38-transfected cells were selected for further analyses (boxed in red).

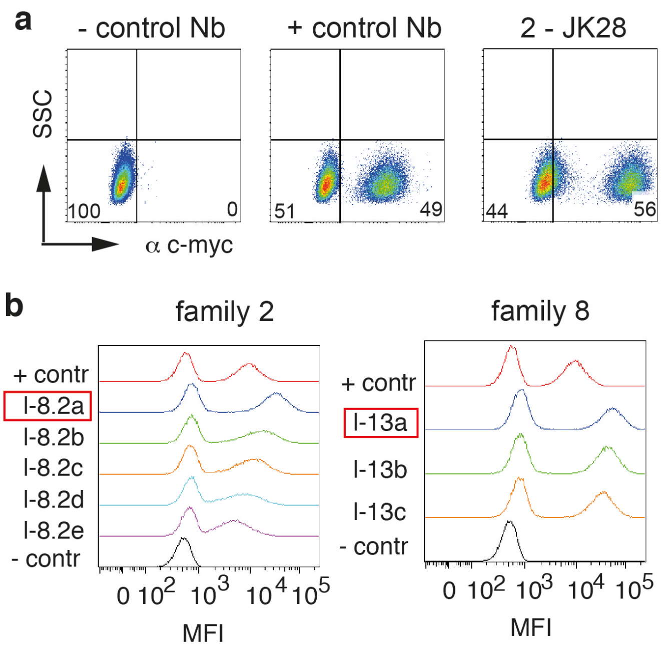

Supplementary Figure S3 | Production of monomeric CD38-specific nanobodies in *E. coli* and HEK-6E cells. (**a**) Proteins in periplasmic lysates (PPL) of IPTG-induced *E. coli* cells were analyzed by SDS-PAGE and Coomassie staining. Each lane was loaded with 20 µl of periplasmic lysate derived from 1 ml of *E. coli* culture. (**b**) HEK-6E cells were transfected with expression constructs for CD38-specific nanobodies and grown in serum free medium for 6 days. Proteins in cell supernatants (10 µl/lane) were analyzed by SDS-PAGE and Coomassie staining.

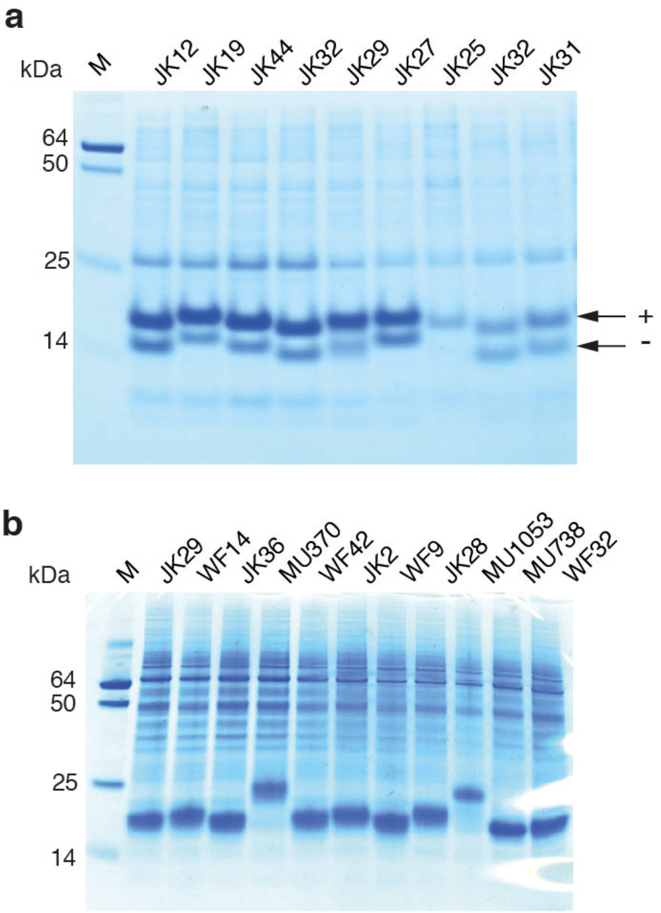

Supplementary Figure S4. Fluorochrome-conjugated Nanobodies slowly dissociate from cell surface CD38. CD38-transfected Yac-1 cells were incubated with excess (100 nM) Alexa^{647}-conjugated nanobodies for 30 min at 4°C. Cells were washed three times and then monitored for loss of cell-associated fluorescence over time at 4°C. An equal aliquot of CD38-expressing cells that had been labeled with the cell-tracking dye eFluor 450 was added at t= 0 as a sink for the dissociated Nbs. Binding of Alexa^{647}-conjugated nanobodies to eFluor450-labled cells is possible only after dissociation from the originally bound (eFluor450-negative) cells.

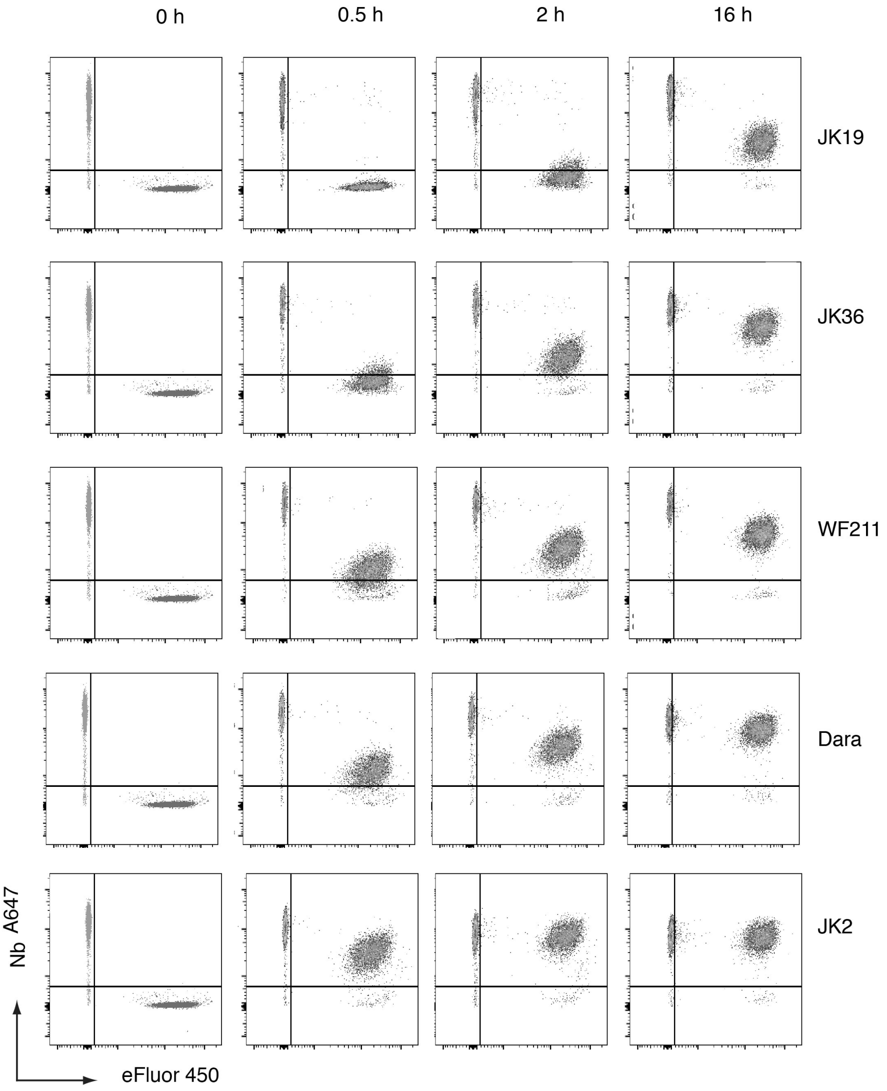

Supplementary Figure S5. Mapping of the epitope specificity of nanobodies by cross-blocking analyses. CD38-transfected Yac cells were preincubated for 30 min at 4°C with unconjugated nanobodies (2 µg/100 µl) before addition of fluorochrome-conjugated nanobodies (0.2 µg). Cells were further incubated for 30 min at 4°C, washed and analyzed by flow cytometry.

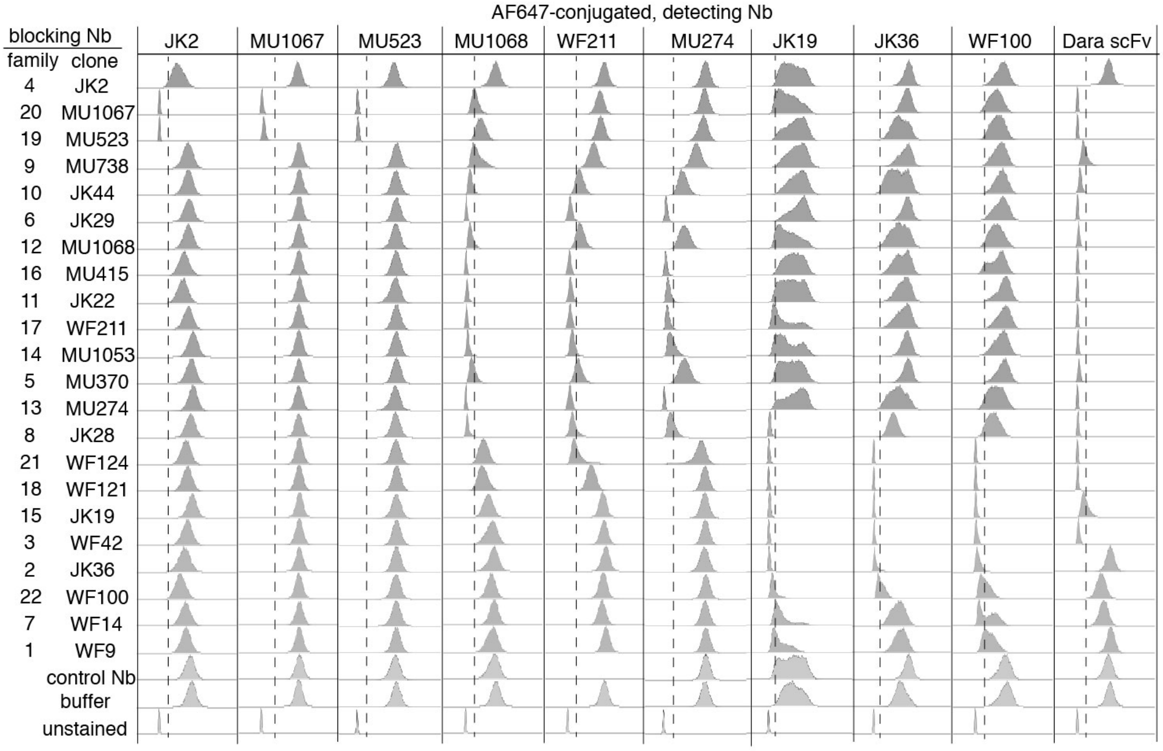

Supplementary Figure S6. Production, purification, and fluorochrome conjugation of monovalent nanobodies and Dara, the single chain variable fragment of daratumumab. **a**) Schematic diagram of monovalent recombinant antibody fragments derived from conventional and llama heavy chain antibodies. **b**) HEK-6E cells were transfected with cDNA-expression constructs encoding single chain variable fragments (scFv) or nanobodies (Nb) fused C-terminally to a His6x tag. Transfected cells were cultured in serum free medium for 6 days. Cell supernatants were clarified by centrifugation and analyzed by SDS-PAGE and Coomassie staining (S, 10 µl supernatant). Dara and Nbs were purified by affinity chromatography on Ni-NTA. Proteins were separated from imidazole by size-exclusion chromatography on PD10 columns. F: flow through (10 µl of 60 ml), W: wash (10 µl of 20 ml), E1: first eluate (10 µl of 1.25 ml), E2: second eluate (2.5 µl of 2.5 ml), E3: third eluate (10 µl of 2.5 ml), G: eluate of the PD10 column (3,5 µl of 3.5 ml), P: purified protein adjusted to 2 mg/ml (1 µl). M molecular weight marker proteins , kD = kilodalton. **c**) Purified nanobodies and Dara were incubated for 30 min with Alexa[647]. Fluorochrome-conjugated proteins were separated from unconjugated dye by size exclusion chromatography. Proteins were analyzed by SDS-PAGE and visualized by Coomassie staining; flurochrome-labeled proteins were visualized by imaging of the gel on an IVIS200.

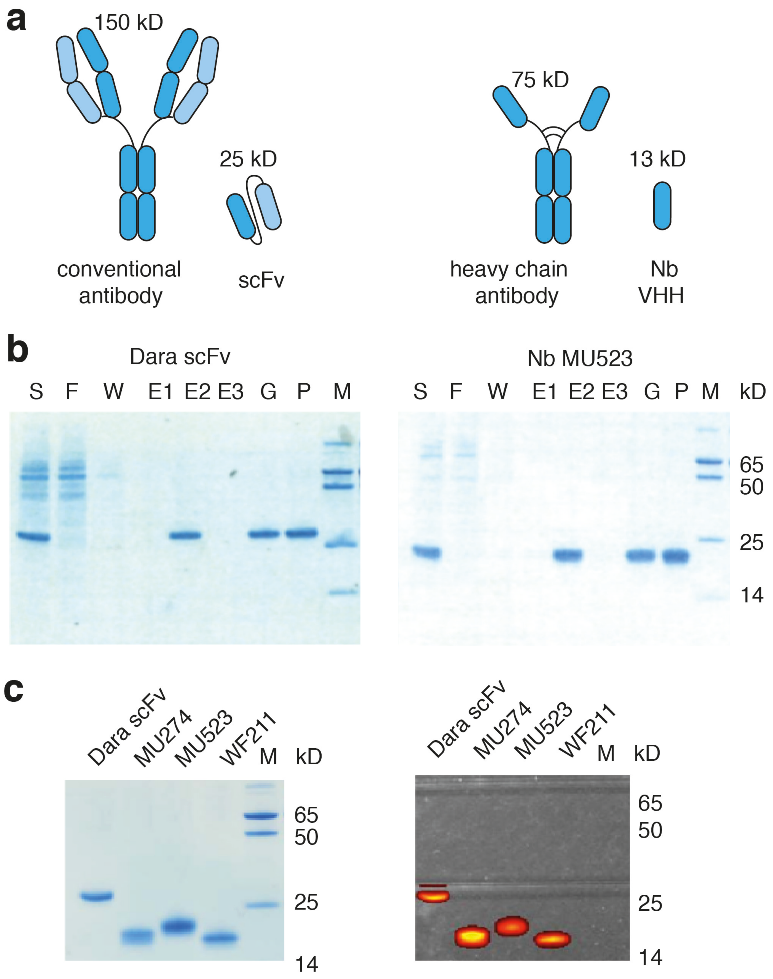

Supplementary Figure S7. Bio-layer interferometry analyses show that nanobodies of families 1, 2, 4 and 22 can bind simultaneously with Dara scFv. Sequential BLI binning analyses of Dara scFv and nanbodies were performed on CD38 immobilized on AR2 Biosensors using the Octet RED384. (**a**) Control BLI analyses with Dara scFv as first analyte followed by either an irrelevant nanobody as negative control or Dara scFv (self-binning control) for second analyte. (b) BLI analyses with Dara scFv as first analyte followed by nanobodies of family 1 (WF9), family 2 (JK36, WF32), family 4 (JK2), and family 22 (MU1105, WF100, WF114) for second analyte.

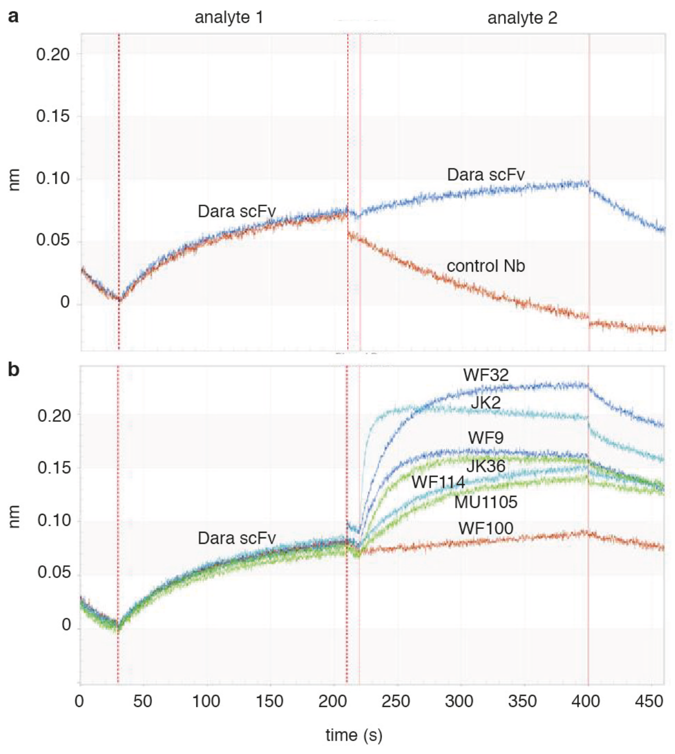

Supplement: Supplementary file 1 — Supplementary Information [file 41598_2017_14112_MOESM1_ESM.pdf]
